# Health- Related Quality of Life Among Jordanian Adolescent Cancer Patients Receiving Active Treatment

**DOI:** 10.31557/APJCP.2019.20.10.3107

**Published:** 2019

**Authors:** Saad Al-Nassan, Noor Al-Bitar, Dhuha Saad, Amani Zahran, Felasteen Elayyan, Shereen Al-Awadi, Kawther Khaleifeh, Anwar Al-Nassan

**Affiliations:** 1 *Department of Physical and Occupational Therapy, Faculty of Applied Health Sciences, The Hashemite University, Zarqa,*; 2 *Department of Pediatric Oncology, King Hussein Cancer Center, Amman, Jordan. *

**Keywords:** HRQOL, adolescent, malignancy, Jordan

## Abstract

**Aims:**

The aim of this study was to evaluate quality of life among adolescents diagnosed with malignancies and currently undergoing treatment in the age group of 13-18 years old.

**Method:**

The study used a descriptive cross-sectional design. Participants were recruited from the pediatric oncology department at King Hussein Cancer Center in Jordan. The Arabic version of self-report (adolescent 13-18) pediatric quality of life inventory (PedsQLTM ) 4.0 Generic Core Scale was used.

**Results:**

Eighty patients were enrolled in the study. The total score of PedsQL 4.0 generic core was 62.0 (SD=16.5). The highest score was for social functioning (mean=85.4, SD=18.4) and the lowest score was school functioning (mean=39.5, SD=28.4). Females had significantly higher scores for health-related quality of life (HRQOL) in school functioning. Type of tumor did not affect HRQOL. Outpatient participants had significantly higher scores for HRQOL in all domains, except in school functioning where inpatients had a significantly higher score.

**Conclusion:**

To the best of our knowledge, this is the first study to reveal the HRQOL scores for Jordanian adolescents with cancer. Addressing the special needs in such a unique age group is essential when planning a comprehensive care plan for a better quality of life.

## Introduction

The World Health Organization defines Quality of Life as an individual’s perception of their position in life in the context of the culture and value systems in which they live and in relation to their goals, expectations, standards and concerns (WHO, 1997). The concept of health-related quality of life (HRQOL) encompasses the overall quality of life that can clearly affect physical and mental health perceptions; this includes health risks and conditions, functional status, social support, and socioeconomic status (Center for Disease Control and Prevention, 2000; Sosnowski et al., 2017). Adolescence is a unique and complex developmental phase characterized not only by significant physical and cognitive changes, but also by critical psychosocial challenges, relating to self-identity, peer relationships, development of autonomy, and sexuality (Sodergren et al., 2017). Compared with children and older adults, adolescence age group is regarded as particularly vulnerable when talking about HRQOL measures (Bleyer, 2005; Thomas et al., 2010). For adolescents with cancer undergoing treatment, their special needs are often not fully addressed; this can easily decrease their overall quality of life after completing the treatment and during adulthood. Examples of special physical needs for adolescents that can be directly affected by cancer and cancer treatment include abilities to combat pain, fatigue, body image and fertility issues (Snobohm et al., 2010). While psychosocial concerns affected by cancer and cancer treatment could include maturing autonomy, values, emotional, and social relationships (Williams, 2013). 

According to the Jordanian Department of General Statistics, Jordanian adolescents contribute about 12.4% of Jordan’s population (Department of Statistics, Jordan, 2015). Cancer among adolescents and young adults represents 16.3% of all new cancer cases in Jordan (Abdel-Razaq et al., 2018). HRQOL has been evaluated in healthy as well as diabetic and obese adolescents in Jordan (Al-Akour et al., 2010; Al-Akour et al., 2012; Hournai et al., 2017). 

The objective of our study was to evaluate HRQOL among Jordanian adolescent cancer patients receiving active treatment. So far, there are no studies that describe the quality of life aspects among Jordanian adolescent cancer patients based on their own perception. The significance of this study relies in addressing the quality of life needs for a sensitive age group of patients who shall soon have active roles in the community as young adults. 

## Materials and Methods

*Participants*

The study adopted a cross-sectional descriptive design, in which adolescent cancer patients ageing between 13-18 years old were recruited through the pediatric oncology department at King Hussein Cancer Center. This specialized cancer center located in Amman, the capital city of Jordan, provides cancer treatments for patients from all over Jordan and from neighboring countries. Inclusion criteria included Arabic-speaking males and females, ageing between 13-18 years old, diagnosed with a malignant tumor by medical oncologists at KHCC and currently under treatment for at least 1 month, either in the inpatient or the outpatient pediatric units. Exclusion criteria included patients who were unable to communicate with the investigators due to physical, mental or cognitive disorders, or could not understand the questions of the investigator during the interview. 

*Data collection*

Recruiting eligible patients was done by trained research assistants (RAs) working at KHCC. The study questionnaire was administered using private face-to-face interview in a private room for the participants from the inpatient and outpatient units. The interview included two main parts: the first part included filling basic information by the RA using the patient’s medical records, asking the patient about his/her date of birth, school grade, and if he/she could read the questionnaire. The second part included filling the questionnaire by the patient. The RA explained the questionnaire items and the scale, and filled the questionnaire on behalf of patients who were unable to read or write but could understand the question and expresses the responses.

*Instrument*

The Arabic version of the Pediatric Quality of life Inventory (PedsQL 4.0) was used to assess the quality of life in adolescents. The PedsQL consists of developmentally appropriate forms of child self-report for the age groups 5-7, 8-12, 13-18 and parent proxy-report for ages 2-4, 5-7, 8-12, 13-18. The scale was developed by Varni et al. (2003). The PedsQL 4.0 Generic Core Scales consists of 23 items applicable for healthy school and community populations, as well as pediatric populations with acute and chronic health conditions. The generic module comprises of twenty-three items that contribute for four subscales: physical functioning (8 items), emotional functioning (5 items), social functioning (5 items) and school functioning (5 items). A five-point response scale is used. The instructions ask how much of a problem each item has been during the past one month. The response scale for each item is “never” (0), “almost never” (1), “sometimes” (2), “often” (3), and “almost always” (4). Responses are then transformed to 100, 75, 50, 25, and 0, respectively, resulting in a range of 0-100, so that higher scores indicate better health-related quality of life (Varni and Limbers, 2009). The PedsQL 4.0 Generic Core scales have shown high reliability and validity in both healthy and patient pediatric populations (Varni et al., 2002; Varni and Limbers, 2009; Arabiat et al., 2011; Roberts et al., 2012;). An Arabic version of the PedsQL 4.0 Generic Core scales (self-report and parent proxy-report) was validated and approved by Arabiat et al., (2011), the authors found satisfactory psychometric properties of the Arabic version of the scale that allows for QOL measurement among the target age group. A permission of use was obtained from original owner of the questionnaire: “PedsQL™ contact information and permission to use: Mapi Research Trust, Lyon, France – Internet: https://eprovide.mapi-trust.org and www.pedsql.org/index.html ”. 

The IRB committee at King Hussein Cancer Center has approved the study under the No. 16 KHCC 27. The investigators of the study have approached the parents of participating patients; explained the objectives and procedures of the study, and obtained their assents. 

*Statistical analysis*

Statistical analysis was done using SPSS software version 21. For descriptive analyses of clinical characteristics, means, standard deviation (SD), frequencies (N), and percentages were used. Independent sample t-tests were used to evaluate differences in quality of life scores between males vs. females, leukemia and lymphoma vs. solid tumors, and inpatients vs. outpatients. A p-value of ≤ 0.05 was considered to be statistically significant. 

## Results

*Sample characteristics*

A total of 80 patients were interviewed in the study. Table 1 gives demographic and clinical characteristics of the patients. There were 55% males, 45% females, and the mean age was 15.6±1.82 years. The majority of patients (63.8%) were receiving chemotherapy treatment. 

*PedsQL 4.0 generic core scores for all participants*

Table 2 shows the mean scores of the PedsQL 4.0 for all participants (n=80). The total score mean was 62.0 (SD=16.5), while the highest subscale score was for social functioning with a mean score of 85.4 (SD=18.4), and the lowest subscale score was for school functioning with a mean score of 39.5 (SD=28.4). The mean score for the psychosocial functioning was 62.1 (SD=16.5). 

*PedsQL 4.0 generic core scores for male vs. female participants*

The eighty participants in the study were divided into 2 groups of males and females. We had 44 (55%) males and 36 (45%) females. Table 3 shows the mean scores of the PedsQL 4.0 for male vs. female participants. A significant difference was found in the school functioning subscale between males and females, as females scored a higher school functioning score with a mean of 49.4 (SD=24.6) while males had a mean score of 31.4 (SD=29.0). 

*PedsQL 4.0 generic core scores for patients with leukemia and lymphoma vs. patients with solid tumors*

We classified the participants into 2 subgroups based on their primary diagnosis. Thirty-four patients (42.5%) had leukemia or lymphoma, while 46 (57.5%) patients had solid tumors. Table 3 shows the mean scores of the PedsQL 4.0 for leukemia and lymphoma vs. solid tumor patients. No significant differences were found between the two groups. The means for total scores were 63.6 (SD=13.4) and 60.7 (SD=18.6) for leukemia and lymphoma group and solid tumors group, respectively. Both groups had the highest scores in the social functioning subscale, while the lowest scores were in the school functioning subscale.

*PedsQL 4.0 generic core scores for inpatients vs. outpatients*

In order to know the impact of admission status on the quality of life of adolescent patients, we sub-grouped the participants into 2 main subgroups based on their admission status. Thirty-three patients (41.3%) were hospitalized and receiving treatment as inpatients, and 47 (58.8%) were receiving treatment in the outpatient clinics and wards as outpatients. Table 4 shows the scores for the total and other subscales. The outpatient group scores were significantly higher than inpatient scores in all domains except for the school functioning, where the inpatient group score was significantly higher. Both groups scored highest in social functioning; the means were 75.3 (SD=20.9) and 92.6 (SD=12.2) for the inpatient group and outpatient group, respectively. The lowest scores recorded for the inpatient group were in the physical functioning [Mean= 47.6, SD=24.5] and school functioning subscales [Mean=47.7, SD=28.2]. The outpatient group scored lowest in the school functioning subscale with a mean of 33.7 (SD=27.4). 

## Discussion

The current study aimed to evaluate HRQOL among Jordanian adolescent cancer patients receiving active treatment. The study compared HRQOL scores according to gender, type of tumor, and admission status, using the Arabic version of PedsQL 4.0 Generic Core Scale. 

Our results showed that participating adolescents scored highest in social functioning subscale, and lowest in school functioning subscale. This agrees with the results of Arabiat et al. on cancer and chronically ill Jordanian children ageing between 6-16 years old (Arabiat et al., 2011). HRQOL scores for healthy adolescents in Jordan show similar tendency as scores were higher in the social domains, indicating better quality of life in social aspects of life (Hourani et al., 2017). Our results also concur with results from Turkish population using the same investigation instrument (Arslan et al., 2013). We propose this tendency is due to the strong cultural and traditional structures in Middle Eastern communities, where family and society support to children and adolescents is enormous. 

**Table 1:** Patients Characteristics (n=80)

Age mean (SD)	N	%
	15.6 (1.82)	
Gender		
Male	44	55
Female	36	45
Diagnosis		
Leukemia and Lymphoma	34	42.5
Solid tumor	46	57.5
Admission status		
Inpatient	33	41.3
Outpatient	47	58.8
Type of active treatment		
Radiotherapy	4	5
Chemotherapy	51	63.8
Surgical	2	28.8
Combined	23	2.5

**Table 2:** PedsQL TM 4.0 Generic Core Scores for All Patients (N=80)

Variable	Mean ± SD
Total score	62.0 ± 16.5
Physical functioning	61.5 ± 26.8
Emotional functioning	61.4 ± 24.4
Social functioning	85.4 ± 18.4
School functioning	39.5 ± 28.4
Psychosocial score	62.1 ± 16.2

**Table 3:** PedsQL TM 4.0 Generic Core Scores (Male Vs. Female)

Variable	Male (N=44) Mean ± SD	Female (N=36) Mean ± SD	t-test P value
Total score	61.1 ± 16.3	62.9 ± 16.9	0.6
Physical functioning	64.6 ± 27.1	57.6 ± 26.2	0.2
Emotional functioning	62.6 ± 24.3	60.0 ± 24.8	0.6
Social functioning	85.9 ± 18.7	84.9 ± 18.2	0.8
School functioning	31.4 ± 29.0	49.4 ± 24.6	0.004
Psychosocial score	59.9 ± 16.2	64.8 ± 16.3	0.2

**Table 4:** PedsQL TM 4.0 Generic Core Scores (Leukemia and Lymphoma Vs.Solid)

Variable	Leukemia & Lymphoma (N=34) Mean ± SD	Solid (N=46) Mean ± SD	t-test P value
Total score	63.6 ± 13.4	60.7 ± 18.6	0.4
Physical functioning	66.7 ± 27.5	57.6 ± 25.8	0.1
Emotional functioning	64.3 ± 20.6	59.4 ± 26.9	0.3
Social functioning	88.5 ± 12.9	83.2 ± 21.4	0.2
School functioning	35.0 ± 31.4	42.8 ± 25.9	0.2
Psychosocial score	62.6 ± 13.9	61.8 ± 18.0	0.8

**Table 5:** PedsQL TM 4.0 Generic Core Scores (Inpatienet Vs. Outpatient)

Variable	Inpatient (N=33) Mean ± SD	Outpatient (N=47) Mean ± SD	t-test P value
Total score	55.5 ± 18.1	66.5 ± 13.8	0.003
Physical functioning	47.6 ± 24.5	71.1 ± 24.1	< 0.0001
Emotional functioning	51.2 ± 25.6	68.6 ± 21.0	< 0.001
Social functioning	75.3 ±20.9	92.6 ± 12.2	< 0.0001
School functioning	47.7 ± 28.2	33.7 ± 27.4	0.03
Psychosocial score	58.1 ± 19.0	65.0 ± 13.7	0.06

Our study did not show significant differences between males and females for physical functioning or psychosocial summary scores, but a significant difference was found between males and females in school functioning indicating a better quality of life in school functioning domain in female patients. Although a study on HRQOL in Jordanian adolescents with overweight or obesity revealed that overweight/obese females had poorer QOL in all domains (Al-Akour et al., 2012), studies on gender differences in school performance among adolescents diagnosed with cancer or other chronic illness are scarce. A study on quality of life in healthy Jordanian adolescents did not find significant differences between males and females on the school environment domain (Hourani et al., 2017). However, a study by Klatchoian et al. on Brazilian children found that healthy female adolescents scored higher in the school dimension (Klatchoian et al., 2010). 

In the current study the primary cancer site did not influence HRQOL among adolescents. The social functioning subscale was highest and school functioning subscale was lowest in patients with leukemia, lymphoma, and solid tumors. Studies on the impact of cancer diagnosis on HRQOL have conflicting results, with some studies showing no correlation between type of cancer and HRQQL of children, and others showing only little impact of cancer diagnosis on HRQOL (Eiser et al., 2005; Sung et al., 2009; Barakat et al., 2010; Erickson et al., 2011; Kuhlthau et al., 2012). A study by Hinds et al. reported that children with Acute Lymphoblastic Leukemia (ALL) have higher HRQOL than children with solid tumors (Hinds et al., 2009), while another study reported that children with ALL experience lower HRQOL than other cancer diagnoses (Landolt et al., 2006). These conflicting results make it harder to rule out whether the primary site of cancer has an impact on HRQOL. 

We conducted a comparison between participants by dividing them according to their admission status. Outpatient participants reported significantly higher HRQOL scores in all domains except for school functioning, where inpatient participants had a significantly higher score than outpatients. Hospitalization days in addition to illness and treatment burdens can exert a great deal of physical and psychological distress on children (Ami, 2016). Although studies on the impact of hospitalization on the quality of life of adolescents are limited, our findings are consistent with published literature (Landolt et al., 2006; Sung et al., 2009). Two studies showed that both child and parent reported a higher HRQOL when treated in a home environment as compared to a hospital environment (Stevens et al., 2006; Speyer et al., 2009). In children with cancer, treatment can prevent many children from attending school full time, causing them to miss out in important academic and social learning opportunities (Brand et al., 2017). The Back to School Program conducted at King Hussein Cancer Center where the data was collected, enables inpatient children undergoing treatment to continue their school functions through the help of teachers from the ministry of education. We assume that outpatient participants had more days of absence from their schools while undergoing outpatient treatment and follow-up, and/or due to other medical complications, which might have affected the quality of their school life significantly in comparison to their inpatient peers. 

Our study is not without limitations, the cross-sectional design for data collection allows only for timely limited observation on HRQOL of participants in the past month. In addition to this limitation, our study did not correlate the length of hospitalization period to HRQOL in adolescents. This implies further investigations in future studies. Despite the mentioned limitations, our study revealed for the first time in Jordan, some important comparisons, and basic descriptive statistics for HRQOL for an age group that have special characteristics and needs.

In conclusion, adolescent cancer patients undergoing active treatment have compromised HRQOL on both physical and psychosocial aspects. Cancer treatment for adolescents and young adults (AYA) is a fast growing specialty in oncology and this population should be recognized as separate. Addressing the HRQOL needs for this age group is essential in order to establish a comprehensive plan of care that is well customized to their needs. 
